# Bacterial adherence: much more than a bond

**DOI:** 10.3934/microbiol.2018.3.563

**Published:** 2018-07-11

**Authors:** Patrick Di Martino

**Affiliations:** Groupe Biofilm et Comportement Microbien aux Interfaces, Laboratoire ERRMECe-EA1391, Université de Cergy-Pontoise, rue Descartes site de Neuville-sur-Oise 95031 Cergy-Pontoise, cedex France

Bacterial adherence is the beginning of the process of colonization of a surface known as biofilm development that involves physicochemical and molecular interactions [1]. Thus, adhesion to inert surfaces is generally related to nonspecific interactions and adhesion to biological surfaces is related to a specific ligand-receptor interaction [2],[3]. The receptor specifically targeted for bacterial adherence can be a protein or a glycoconjugate expressed on the surface of host cells, or present in connective tissue or adsorbed on an abiotic surface in contact with host tissues. In Gram-negative bacteria, adherence factors can be classified as fimbrial, non-fimbrial, and discrete polysaccharide adhesins [2]. Fimbrial adhesins also called pili form polymeric protein fibers. Pili are multifunctional appendages that can be involved in attachment to and motility on surfaces, immunomodulation, biofilm formation, DNA transfer, and electron transfer. In Gram-positive bacteria, pili have been described more recently in pathogenic and non-pathogenic bacteria [4]. One particular feature of pili in Gram-positive bacteria is the use of covalent links between fimbrial subunits to produce the pilus fiber. In addition to proteinacous adherence factors, many bacterial species produce extracellular polysaccharides involved in adhesion [2]. The extracellular polysaccharide adhesin termed poly-N-acetylglucosamine (PNAG) or polysaccharide intercellular adhesin (PIA), is involved in Staphylococcal adherence to abiotic surfaces and biofilm formation [5],[6]. “Microbial surface components recognizing adhesive matrix molecules” (MSCRAMMs) are surface proteins expressed only by Gram-positive bacteria [7]. MSCRAMMs mediate bacterial adherence to the extracellular matrix molecules like collagen, fibrinogen, fibronectin, or laminin. Fibronectin-binding proteins (FnBPs) are surface adherence factors expressed by both Gram-positive and Gram-negative bacterial pathogens and commensals [3],[8]. The denomination FnBP is based only on functional properties since no common sequence features have been identified among this large family of molecules.

Lactic acid bacteria (LAB) include several species of Gram-positive bacteria that live in close association with humans [9]. Adherence properties of LAB to epithelial surfaces of the host are a key element in their probiotic activity. Probiotics are defined as “live microorganisms that, when administered in adequate amounts, confer a health benefit on the host” [10]. The close association between bacteria and epithelial cells is essential for maintaining LAB in the host and stimulating local immune defense [11]. Pili have been described in LAB like *Lactobacillus rhamnosus*, or *Lactococcus lactis* [12],[13] and different types of surface molecules (carbohydrate, proteins, and lipids) seem to be involved in the adhesion of LAB to epithelial cells [14].

In addition to adherence to a different surface, bacteria can adhere each other to form clumps and microcolonies. Some bacteria of the same type form multicellular clumps by self-recognising surface structures, such as exopolysaccharides or proteins [15]. Proteinaceous autoagglutinins involved in this process include non-fimbrial adhesins, flagella and pili. The family of autotransporters of Gram-negative bacteria include many autoagglutinins [16]. This phenomenon called autoaggregation can be among the initial steps of biofilm formation by participating in the formation of microcolonies. After attachment of isolated planktonic cells to a surface, other cells in suspension attach to the adherent cells via autoagglutinins, leading to the formation of microcolonies. The grouping on a surface of several adherent bacteria thanks to their twitching motility is also linked to autoagglutinins. Some bacteria can also autoaggregate in solution, and the aggregates attach to the substrate as pre-existing microcolonies. In addition to being an initial step of biofilm formation, autoaggregation is a protective mechanism from external stresses such as nutrient starvation or oxidative stress used by both environmental and pathogenic bacteria.

Extracellular polymeric substances, also known as EPS, are mainly polysaccharides, proteins, extracellular DNA and lipids involved in bacteria interactions with their environment. EPS are a key element in understanding the biofilm phenotype [17]. As noted above, EPS may have an initial attachment function of the cells to the colonized substrate, but they may also be involved in later phases of biofilm formation. Non-covalent bonds between EPS through weak physicochemical forces ensure the stability of the matrix. EPS form a network that interacts with bacterial aggregates, provides cohesion, viscoelasticity, and protection to the biofilm.

In an infectious context, adherence to tissues is at the centre of the cross-talk between the pathogenic bacterium and its host [18]. After the adhesion step, bacteria act on the behaviour of the host by modifying its environment and disrupting the immune response. This goes for altering the signalling of host cells to facilitate bacterial survival and spread. Many molecules bound to the surface or secreted by commensal, probiotic, or pathogenic bacteria participate in the colonization of the host. Bacterial adherence factors are usually cell surface structures specialized in the development of interactions with surfaces like Pili, or MSCRAMMs. Some proteins involved in bacterial adherence have been first described as ubiquitous intracellular enzymes referred to as “housekeeping enzymes” but also to as “moonlighting proteins” [19]. Moonlight proteins have two very different functions, like intracellular chaperone or metabolic activity and adhesion, often in two different subcellular locations [19].

In the context of the development of antibiotic resistance, targeting of bacterial virulence and in particular bacterial adherence is a promising anti-infectious strategy [20]. The classic anti-infective strategy is to slow down growth or to induce the decay of the pathogen population. This antimicrobial activity induces a selection pressure that favours the emergence of resistant variants and the dissemination of resistance genes between species sometimes very different. Targeting bacterial adherence may allow infection attenuation and natural clearance. This strategy is based on the use of cocktails of soluble analogues of cellular and tissue receptors of bacterial adhesion factors [21],[22]. In the context of the fight against biofilms, in medicine, in the cosmetics or food industry, another innovative anti-adherence strategy is emerging. It is based on the use of pilicides [23],[24]. An anti-adhesive activity can be obtained thanks to the immobilization on a surface of virstatin, a molecule able to inhibit type IV pili expression. Nevertheless, the inhibition of bacterial adherence associated with the pilicide activity is strain dependent, and virstatin is suspected to increase production of other kind of pili conducting to the induction of interactions between bacteria [24]. Pilicides belonging to the family of bicyclic 2-pyridones active against type I pili expressed by *Enterobacteriaceae* have been shown to inhibit pilus biogenesis of uropathogenic bacteria [25]. Another family of anti-colonization compounds corresponds to inhibitors of amyloid aggregation [26],[27]. Interestingly, such small molecules inhibit biofilm formation but can also disrupt preformed biofilms.

Bacterial adhesion is a natural process that will continue to be studied for a long time, either to deepen its understanding or to control it in a context of sessile biomass use in biotechnology or in a context of fight against bacterial contamination, bacterial infection and biofilm development.
